# Spinal muscular atrophy type 1 in the Caribbean: the first case report from the Dominican Republic

**DOI:** 10.3389/fnins.2024.1476977

**Published:** 2025-01-08

**Authors:** María Belén Martín-Sanz, Delvis Lucas-Muñoz, Manuel Colomé-Hidalgo

**Affiliations:** ^1^Research Group of Humanities and Qualitative Research in Health Science, King Juan Carlos University, Alcorcón, Spain; ^2^Hospital Pediátrico Dr. Hugo Mendoza, Santo Domingo, Dominican Republic; ^3^Universidad Autónoma de Santo Domingo, Santo Domingo, Dominican Republic

**Keywords:** spinal muscular atrophy (SMA), type I, SMN1, Werdnig Hoffmann disease, motor neuron disease, progressive muscular atrophies

## Abstract

Spinal muscular atrophy (SMA) is a progressive genetic neuromuscular condition affecting spinal motor neurons. The underlying cause of SMA is deletions or mutations in the SMN gene. It is classified into five variants based on age and clinical manifestations of the patient. In this report, we present the case discovery of a four-month-old male patient with SMA type 1, presenting with generalized hypotonia and regression of acquired neurodevelopmental milestones. Our study aims to illustrate, through a case report, the clinical analysis, therapeutic interventions, and progression until the patient’s demise. This aims to share the challenges in managing such patients and the strategies employed in their care plan. By documenting this case, our goal is to contribute to the understanding of SMA type 1 and emphasize the ongoing need for learning effective care strategies.

## 1 Introduction

Spinal muscular atrophy (SMA) is a rare progressive genetic neuromuscular disorder affecting spinal motor neurons, caused by defects in both copies of the SMN1 gene, leading to degeneration of alpha motor neurons in the anterior horn of the spinal cord (Masson et al., 2021; Day et al., 2022; Verhaart et al., 2017). SMA is an autosomal recessive neurodegenerative disease, with homozygous mutation on chromosome 5q being the primary genetic cause of infant mortality in the absence of treatment (Day et al., 2022; Eugenio Mercuri et al., 2018). Its incidence is estimated between 1 in 6,000 to 1 in 10,000 newborns (Verhaart et al., 2017; Eugenio Mercuri et al., 2018; Chen et al., 2020). Clinically, hypotonia is the initial symptom in 60% of SMA cases (Pera et al., 2020). Clinical presentations include weakness and atrophy of proximal muscles, areflexia, paradoxical breathing pattern, gastrointestinal dysfunction, metabolic disturbances, orthopedic complications, cognitive impairments, and deficits in social interaction and expression (Masson et al., 2021; Day et al., 2022; Gusset et al., 2021; Aponte Ribero et al., 2023; Corsello et al., 2021; Saladini et al., 2020). Severe cases result in severe mobility limitations and ventilatory insufficiency that can lead to patient mortality (Day et al., 2022).

Four subtypes of SMA (Type 0, I, II, III, IV) have been described based on age of onset, clinical severity, and life expectancy (Giannotta et al., 2024; Schorling et al., 2020). Within the SMA subtype classification, this case corresponds to Type I, also known as Werdnig-Hoffmann disease or severe SMA. This subtype manifests within the first 6 months of life and carries a mortality rate of 50% by 1 year of age, with a survival rate of 8% at 20 months (Gregoretti et al., 2013; Lally et al., 2017). Addressing this condition requires new paradigms for multidisciplinary coordination to maximize autonomy and address multiple healthcare needs, alongside updated knowledge of healthcare interventions for these patients (Finkel et al., 2018; Perego et al., 2020; Bach et al., 2024).

Herein, we present the clinical findings, intervention, and evolution of what, to our knowledge, is the first documented case of SMA Type 1 detected in the Dominican Republic. Despite initial clinical presentation and applied treatment, the patient succumbed at 6 months of age.

## 2 Patient information and clinical findings

The male patient debuted at 3 months of age with generalized hypotonia, impaired mobility, and regression in previously acquired neurodevelopmental milestones. Regarding perinatal history, the child was born via cesarean section at 38 weeks to a 30-year-old mother. This was her third pregnancy and third cesarean section. The patient was admitted after a three-day history of fever accompanied by 2 days of respiratory distress, diagnosed with bronchial syndrome and community-acquired pneumonia.

**Figure 1:**
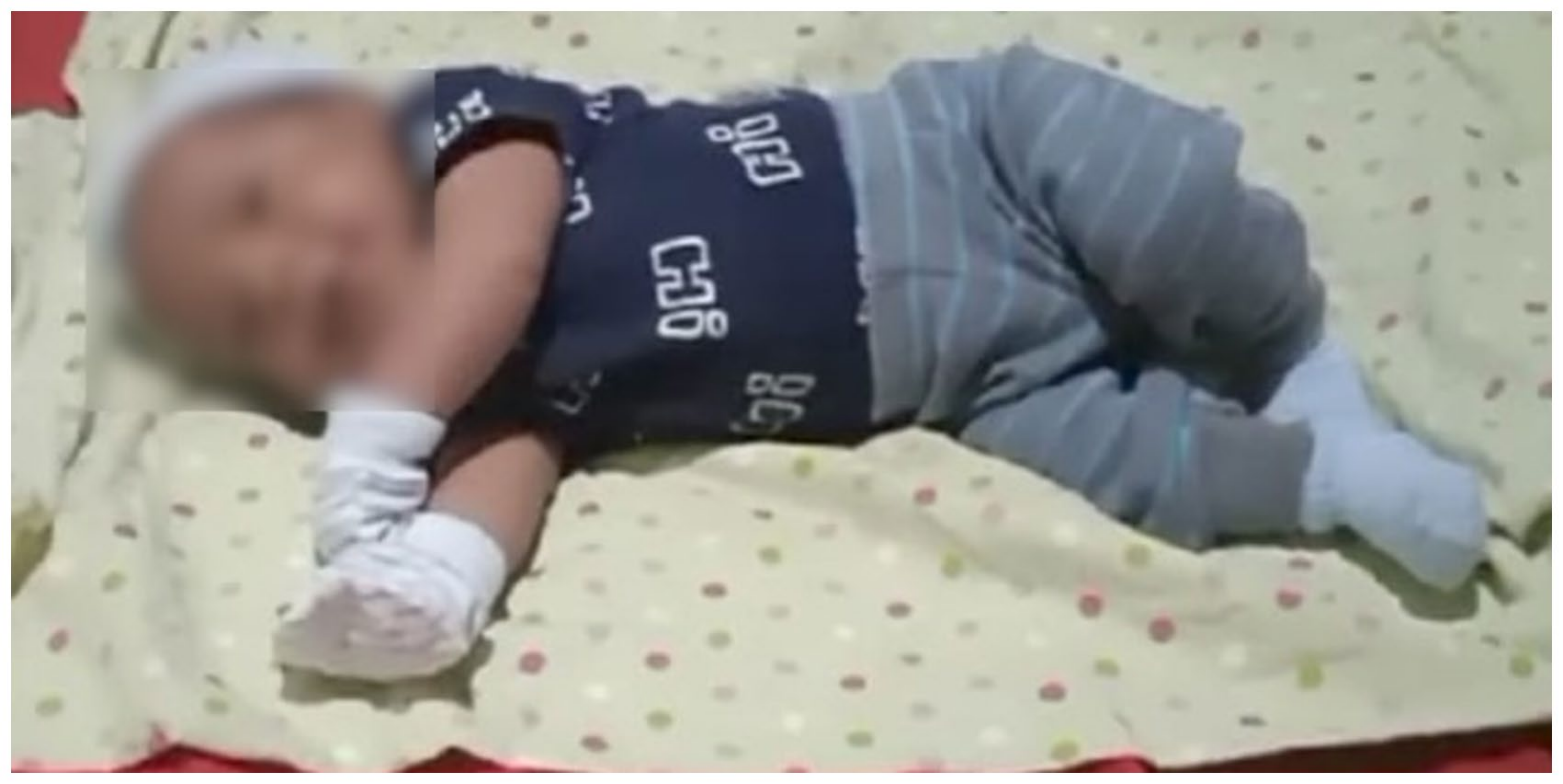
Generalized hypotonia observed in the patient.

Three weeks later, he returned for outpatient consultation requested by his parents at the pediatric neurology service due to concerns about symptoms like those presented by a deceased male sibling at 3 months of age 2 years earlier (loss of crying, generalized hypotonia, and loss of mobility). The parents reported that the sibling died from cardiopulmonary arrest after a month in intensive care, necessitating tracheotomy, with no confirmed differential diagnosis. In Figure 1, a photo of the patient depicts trunk and limb weakness, loss of head control, and inability to roll over. There are no known cases in other family lines.

In this physical examination, the patient is hypoactive, afebrile, eupneic, and well-hydrated. He presents with a symmetrical and normodynamic thorax, although subcostal retractions are noted. Oral cavity lesions, cough, and rhinorrhea were observed in this patient, findings commonly associated in such cases with bulbar muscle weakness due to impaired secretion clearance and increased risk of secondary respiratory and oral infections. Despite the presence of crackles, rhonchi, and crepitations, lung ventilation appears adequate with audible vesicular breath sounds. Cardiac rhythms are regular with no audible murmurs. The abdomen is flat, depressible, non-tender on palpation, without palpable masses or organomegaly. Hemodynamically, the patient is stable. Upper and lower extremities are symmetrical without edema, and peripheral pulses are normal. There are no allergies reported, and the patient has completed the appropriate vaccination schedule for his age. Neurologically, the patient scores 7 points on the Hammersmith Infant Neurological Examination scale and 3 points on the Children’s Hospital of Philadelphia Infant Test of Neuromuscular Disorders (CHOP INTEND) for motor function evaluation. Genetic testing has been requested due to the patient’s current clinical history and family background, confirming a diagnosis of SMA type 1.

**Figure 2:**
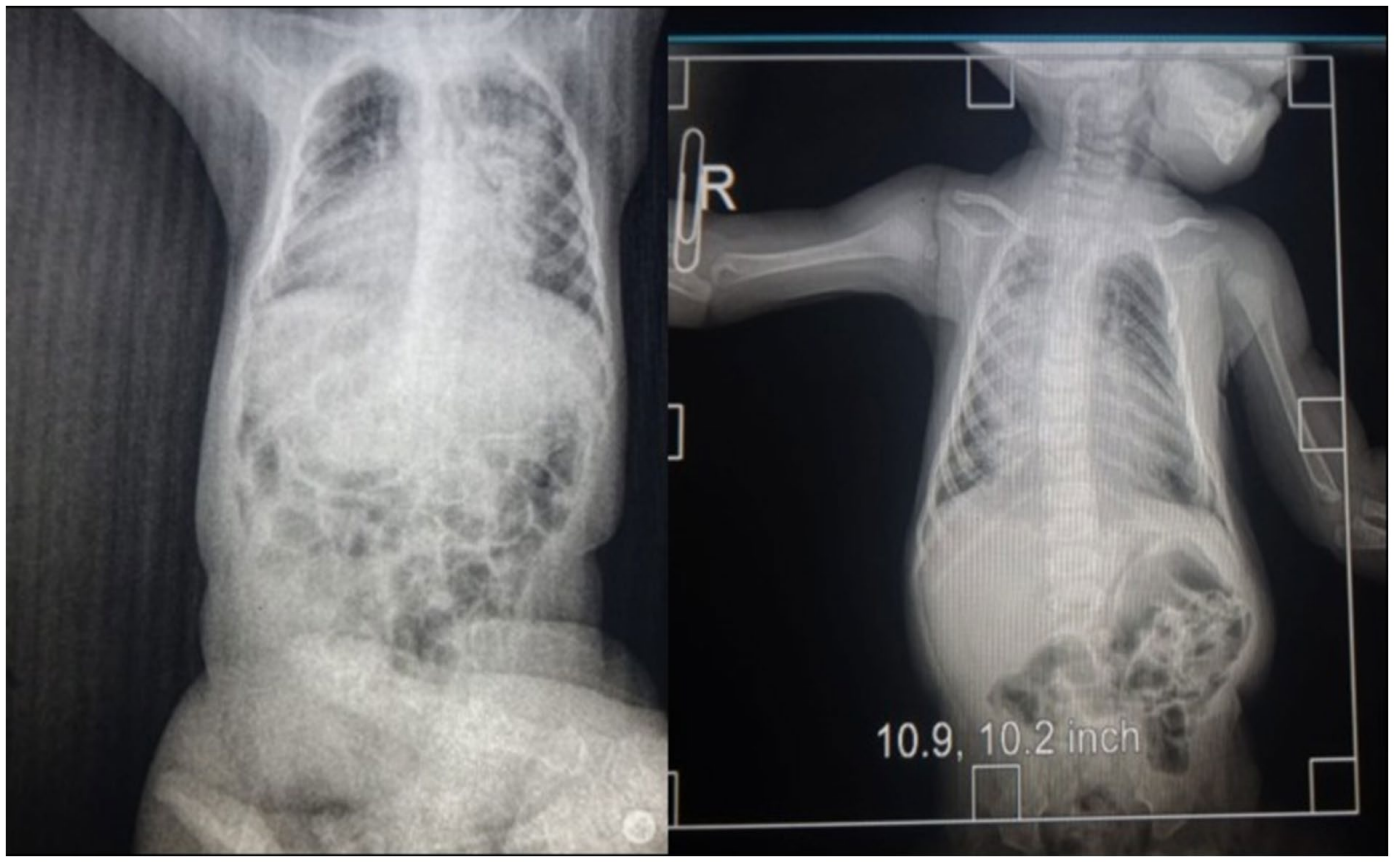
Radiology image.

At 4 months of age, the patient was readmitted urgently due to severe respiratory distress. Parents reported significant difficulty swallowing the previous day and a two-day history of fever. The patient exhibited pallor of the skin and mucous membranes, with centrally and peripherally strong and intense pulses and a capillary refill of 4 sec while receiving dobutamine support at 10 mcg/kg/min. Blood pressure was 115/65 mmHg, and heart rate was 113 beats per minute. Ventilation-wise, audible vesicular breath sounds were present, signs of pneumonia (See Figure 2) under ventilation mode pa/c fio2:80, pins 16, fr:32, and 98% oxygen saturation.

The patient presents with a distended abdomen, visible peristalsis, and palpable hepatomegaly measuring 2 cm. Laboratory tests indicate normal findings. Neurologically, the patient has equal and reactive pupils measuring 3 mm. Cranial CT scan results show no significant abnormalities. Electromyogram could not be performed due to services covered by your insurance.

**Figure 3:**
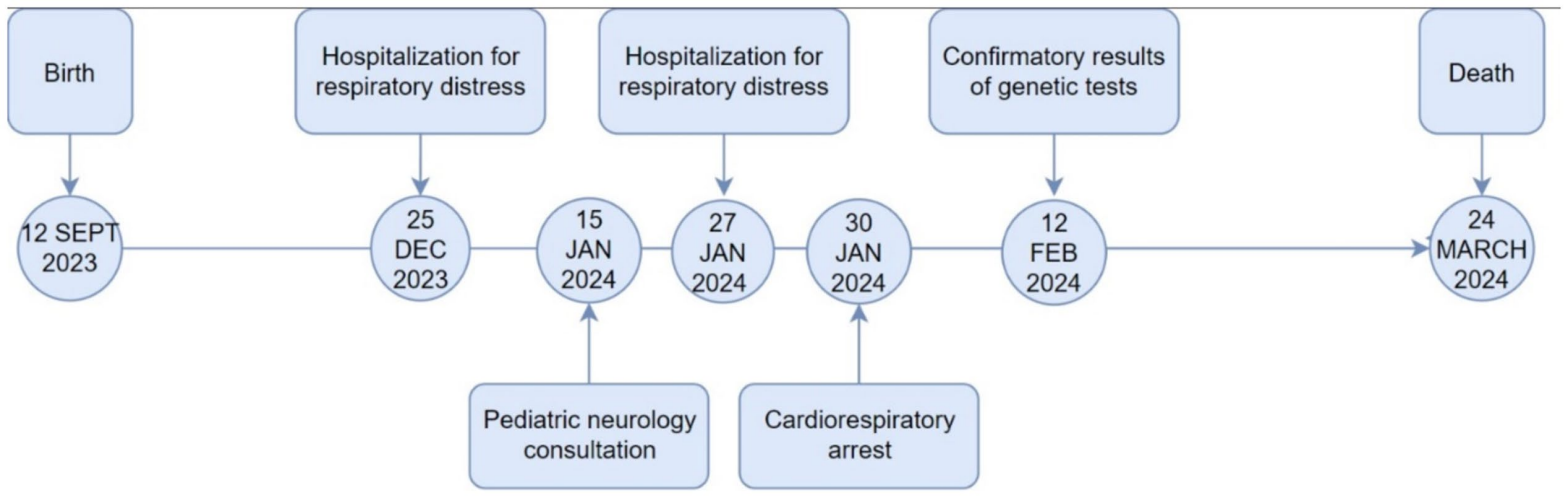
Timeline of key clinical events.

The patient received rescue therapy with salbutamol nebulization (0.15 mg/kg every 5 h, not exceeding 2.5 mg per dose), along with N-acetylcysteine (200 mg every 8 h) and methylprednisolone (1.5 mg/kg, not exceeding 60 mg/day). During the chest X-ray procedure, the patient experienced cardiac arrest, attributed to acute decompensation rather than X-ray exposure. Resuscitation required the administration of three doses of adrenaline. Subsequently, the patient was intubated and transferred to the Intensive Care Unit, where sedation with midazolam and fentanyl was administered. The patient remained in intensive care for 2 months until his death due to cardiorespiratory arrest. In Figure 3, a timeline summary of the patient’s most relevant clinical events is depicted.

## 3 Diagnostic assessment

**Figure 4:**
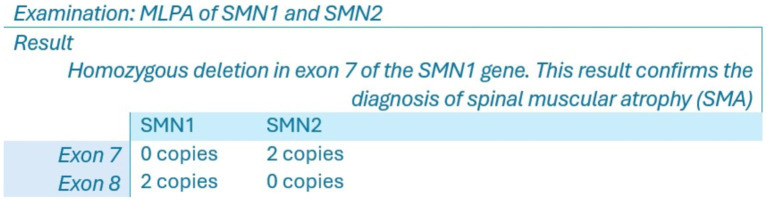
Genetic test results.

Based on the clinical history and findings from the physical examination, a provisional diagnosis of SMA was established. To confirm this diagnosis, genetic testing was performed using a buccal swab sample to extract DNA. The method employed was Multiplex Ligation-dependent Probe Amplification (MLPA), specifically analyzing the copy numbers of exons 7 and 8 of the SMN1 and SMN2 genes. The test had to be requested from a laboratory located outside the country to be conducted in the Dominican Republic. This testing confirmed SMA Type 1 by identifying a homozygous deletion in the SMN1 gene. Figure 4 displays the results of the genetic test conducted on this patient.

## 4 Therapeutic interventions

The treatment prescribed by the Pediatric Neurology Service was Nusinersen, administered intrathecally at a dose of 12 mg/5 mL. Nusinersen is an antisense oligonucleotide that modulates RNA splicing to increase production of the SMN (Survival Motor Neuron) protein. This protein is deficient in patients with SMA due to mutations in the SMN1 gene. The treatment regimen included loading doses on days 0, 14, 28, and 60, followed by a maintenance dose at 4 months. However, this treatment could not be administered due to bureaucratic barriers in obtaining approval for the drug within the country’s High-Cost Medication Program. Approval for administration was granted 15 days after the patient’s death.

## 5 Discussion

SMA presents significant challenges in clinical practice due to its rapid progression and severe complications (Nishio et al., 2023). The intervention followed recommendations from prior studies advocating for an interdisciplinary approach addressing respiratory, nutritional, gastroenterological, orthopedic, and psychosocial issues (Corsello et al., 2021; Kirschner et al., 2020). Management focused on enhancing quality of life and disease progression rather than employing a palliative approach, aligning with previous research findings, which was a key strength in this case (Eugenio Mercuri et al., 2018; Corsello et al., 2021; Finkel et al., 2018; Kirschner et al., 2020). In other patients with SMA, physical training programs lasting at least 12 weeks have been shown to improve muscular and cardiorespiratory function. However, this approach was not implemented in this case due to limited access to specialized physical rehabilitation services (Bartels et al., 2019).

Prescribed treatment included new clinical therapies such as Nusinersen, which has demonstrated improvements in psychomotor function and patient survival rates (Day et al., 2022; Erdos and Wild, 2022; Qiao et al., 2023). Recently, the FDA approved Onasemnogene abeparvovec for SMA type 1, a gene therapy utilizing viral vectors to deliver a functional SMN1 gene copy to motor neurons through a single intravenous infusion (Ogbonmide et al., 2023). Risdiplam has also shown increased overall survival rates in treated patients (Ribero et al., 2022; Ratni et al., 2018). A significant limitation encountered was the accessibility barriers to these treatments in the Dominican Republic. Other proposed interventions suggest the use of gene therapy through a single intravenous injection of an AAV9 vector, which has demonstrated a significant improvement in both life expectancy and motor function in patients. In our case, this clinical approach differed from what was feasible for our patient due to access barriers within the healthcare services of our country (Barkats, 2020). A genotype–phenotype correlation suggests that SMN2 is a potent disease modifier in spinal muscular atrophy (SMA). Recently, two innovative treatments targeting SMN have been approved: antisense oligonucleotides (ASOs) and virus-mediated gene therapy. However, these treatments are not available within the Dominican Republic’s healthcare system, limiting therapeutic options for SMA patients in the country (Chen, 2020).

Another challenge was the inability to initiate presymptomatic treatment, despite literature supporting early screening techniques for timely intervention (Corsello et al., 2021; Institute for Quality and Efficiency in Health Care (IQWiG), 2020; Strauss et al., 2022). Early screening has shown to improve clinical prognosis by addressing muscle weakness in affected patients (Vill et al., 2019), yet difficulties persist in achieving early diagnosis due to limited access to diagnostic evaluations and resource constraints in the region (Pera et al., 2020). Notably, neonatal screening services are not covered by the Dominican Social Security System, thereby creating barriers to accessing high-impact healthcare services necessary for rare disease treatments in our country. High economic costs associated with specific healthcare interventions further strained clinical decision-making and medication administration in this case (Dangouloff et al., 2021). This aligns with previous studies highlighting the economic burden of SMA on healthcare services, negatively influencing decision-making and access to clinical resources (Toro et al., 2023; Yang et al., 2022). Disparities in healthcare access and resources across different geographic areas have been identified, resulting in unmet patient care needs (Paracha et al., 2022; Landfeldt et al., 2021). Such reports help identify gaps in healthcare delivery based on varying healthcare systems, policies, and challenges in implementing internationally established protocols for managing this condition across countries (Farrar et al., 2018).

Moreover, addressing SMA goes beyond clinical treatment to understanding its broader social and familial impacts, as previous studies have shown that having a rare disease significantly affects economic, structural, emotional, and social aspects within families (Dias et al., 2023; Sandilands et al., 2022). This underscores the need for comprehensive biopsychosocial support and assistance for all family members from healthcare resource managers in managing these conditions, emphasizing the importance of increased research in this area (Dias et al., 2023; Sandilands et al., 2022).

In conclusion, documenting unique clinical experiences is crucial for continuous learning and ongoing reevaluation of clinical practice, thereby enhancing understanding and patient care for these conditions.

## Data Availability

The raw data supporting the conclusions of this article will be made available by the authors, without undue reservation.
